# Collocation Use in EFL Learners’ Writing Across Multiple Language Proficiencies: A Corpus-Driven Study

**DOI:** 10.3389/fpsyg.2022.752134

**Published:** 2022-02-09

**Authors:** Xiangtao Du, Muhammad Afzaal, Hind Al Fadda

**Affiliations:** ^1^School of Foreign Languages, Shanghai Jiao Tong University, Shanghai, China; ^2^Institute of Corpus Studies and Applications, Shanghai International Studies University, Shanghai, China; ^3^College of Education, King Saud University, Riyadh, Saudi Arabia

**Keywords:** collocations, foreign language writing, lexical developmental patterns, EFCAMDAT, corpus analysis

## Abstract

The investigation of learners’ interlanguage could greatly contribute to the teaching of English as a foreign language and the development of teaching materials. The present study investigates the collocational profiles of large-scale written production by English learners with varied L1 backgrounds and different proficiency levels. Using the British National Corpus as reference corpus, learners’ collocation use was extracted by corpus query language and further identified by *t*-score *via* Python programming language. The collocation list consists of 2,501 *make/take* + noun (the direct object) collocations. Findings show that proficient learners tend to use collocations containing more semantically complicated and abstract noun elements for varied communication tasks. Moreover, advanced learners are inclined to use collocations comprised of more difficult and longer noun elements.

## Introduction

Collocational competence has been widely recognized as a prerequisite for native-like mastery of target language and attracting substantial attention in the field of language acquisition (Gablasova et al., 2017; Altun, 2021; Cao and Badger, 2021). Along with other types of prefabricated language, collocations help language users economize on cognitive processing effort and reduce dysfluency and hesitation (Fillmore, 1979; Hunston and Francis, 2000). Collocations or “arbitrarily restricted lexeme combinations” (Nesselhauf, 2005), such as *commit crime* or *make a joke,* have been found to take up a large proportion of native speakers’ language production (Cowie, 1992). Based on the investigation of an academic corpus, Howarth (2013) revealed that 41% of verb + noun pairings consist of collocations or idioms. Prefabricated language, including collocation, accounts for approximately half of the spoken and written texts Erman and Warren (2000). Unfortunately, multiple studies have shown that language learners, even those with higher proficiency, have difficulties approximating native speakers’ collocation use (Fan, 2009; Granger and Paquot, 2009; Li and Schmitt, 2010; Yamashita and Jiang, 2010; Laufer and Waldman, 2011). So far, the number of studies investigating learners’ collocation use based on a large scale of longitudinal learner data with multiple proficiency levels is small. The present study set out to address this gap and help clarify the developmental patterns of learners’ productive collocational competence.

## Previous Studies

The past decades have witnessed the surge of research on learners’ collocational competence. Much of the current literature pays particular attention to their productive collocation use in learner corpus. Such studies differ remarkedly in the way they define and identify collocation. Taking a statistical perspective of collocation, some have utilized the frequency-based approach, which is frequently adopted in computational linguistics (Gyllstad, 2007; Nguyen and Webb, 2016; Liu and Afzaal, 2020, 2021). Studies of such kind are highly quantitative (Lee and Shin, 2021) and based on the notion that collocation pertains to the “probability of occurrence of their constituent words” (Henriksen, 2013, p. 31). In contrast to the frequency-based approach, some have adopted the phraseological approach and define collocation by delimiting it from other significant types of combinations, namely, free combinations and idioms, in terms of their degree of transparency and commutability (Nesselhauf, 2005). Aisenstadt (1979) viewed collocation as “combinations of two or more words used in one of their regular, non-idiomatic meanings, following certain structural patterns, and restricted in their commutability not only by grammatical and semantic valency”. Cowie et al. (1988, p. 71) defined collocation by distinguishing it from the other types of multi-word units. Fewer studies have combined the above two approaches to avoid the inconsistency of human judgment in the phraseological approach and the risk of retrieving n-grams which are devoid of meaning, such as *and the* and *by the,* in frequency-based approach (Szudarski and Carter, 2016; Nizonkiza, 2017).

The existing literature also varies greatly concerning their methodology and research design. Most of such studies focused on learners of English from the same first language (L1) backgrounds, for instance, Chinese learners (Li and Schmitt, 2010) and Japanese learners (Yamashita and Jiang, 2010; Saito and Liu, 2021). Moreover, various kinds of measures have been used for the identification of collocations. For example, studies adopting the phraseological approach may rely on native speakers’ judgment (Nesselhauf, 2005), while those taking the frequency-based approach tend to make use of indices of *z*-score, *t*-score, MI score, etc. (Durrant and Schmitt, 2009; Granger and Bestgen, 2014; Gablasova et al., 2017). However, it should be noted that the manual filtering of native-like collocations in learner data is tremendously time-consuming work for either of these two approaches. Researchers taking phraseological approach must apply the relevant criteria, such as transparency and commutability, to identify word combinations one by one. In addition, the frequency-based approach requires the measurement of association strength in each word combination based on their frequency information in large-scale reference corpus. Automatic and reliable identification of the nativelikeness of given word combinations in learner data based on programming language is in need. What is more, a handful of research has assessed the amount and quality of collocation use by intermediate or advanced language learners, with beginners been given insufficient attention (Siyanova-Chanturia, 2015). Studies that managed to investigate a large amount of L2 production by learners at different proficiency levels has been fairly modest until now.

So far, much attention has been accorded to clarifying the deviance in learners’ collocation use by comparing against that of native speakers (Siyanova and Schmitt, 2008; González and Ramos, 2013). Among the various collocation types investigated in previous studies, verb + noun collocations were found to be particularly challenging for language learners (Bahns, 1993; Wang and Shaw, 2008; Tsai, 2015). Laufer and Waldman (2011) found that leaners at all proficiency levels produced significantly smaller number of verb + noun collocations and errors appear to be persistent in fairly advanced learners’ language production. Altenberg and Granger (2001) analyzed EFL learner use of collocations comprised of high-frequency verbs and concluded that this collocation type is surprisingly error-prone and has posed great problems to either beginners or proficient learners. Different reasons for why the uptake of verb + noun collocations is hampered have been proposed. Boers et al. (2014) suggested that high-frequency verb elements in collocations can be problematic as they “contribute relatively little to the semantics of” the collocation as a whole and barely grab learners’ attention. In addition, they summarized that learners may experience particular problems when dealing with semantically related words (e.g., *make* and *do* in *make a mess* and *do damage*) and formally related words (e.g., *make* and *take* in *make a drawing* and *take a photo*). Barcroft (2006) stated that unfamiliar word elements in collocations would hinder learners from mastering the form of collocations by exhausting cognitive processing resources.

Although lots of extant studies focused on the number of collocations accurately used (Siyanova-Chanturia, 2015) or errors (Phoocharoensil, 2014; Kim, 2018) made by learners, studies aiming to identify the properties of learners’ collocation use at different proficiencies are relatively scarce. The following studies may have implications for such a research aim. Peters (2016) investigated the learning burden of different types of collocation in connection with their congruency (presence or absence of literal L1 translation equivalent), collocate-node relationships, and length of constituent words. According to her research, incongruent collocations and verb + noun type collocations tend to cause more difficulty in acquisition. Moreover, collocation items composed of longer words are more challenging to master in a form recall test. Another relevant work was undertaken by Uchida (2015) which highlighted the influence exerted by L2 input. While the factors that could account for learners’ developmental patterns do not seem to be concluding yet, target language input was considered to be crucial for language acquisition and can explain certain acquisition sequences (Rankin and Unsworth, 2016). Reports have shown that second language acquisition is “heavily input-oriented” (Dietrich et al., 1995, p. 271) and input driven (Goldschneider and DeKeyser, 2001). Textbooks in EFL environment are one of the major target language inputs and could greatly influence learners’ acquisition. In Uchida’s research, he analyzed the delexical verb + noun collocations taught in EFL textbooks and proposed that the features of noun elements within collocations (*viz.* semantical fields, concreteness or abstractness, and difficulty levels) may help characterize learners’ collocation use at different proficiencies. Uchida called on further analysis to verify the assumption and explore more properties to profile learners’ collocation use.

Addressing the findings in Uchida’s research, we assume that learners at higher proficiency levels may start to be exposed to collocations made up of more difficult nouns belonging to varied and abstract semantic fields and hypothesize that they tend to use such collocations as proficiency increases. Moreover, referring to Peters’ research, we speculate that collocations containing longer noun elements are better mastered by advanced learners due to its relatively heavier learning burden. Therefore, based on the literature review above, this paper seeks to clarify the characteristics of learners’ productive collocational competence at different proficiency levels. Learners’ collocation use is to be compared in the following aspects, difficulty level, semantic fields, and length of constituent noun elements in the collocation.

This study’s view of collocation is that it can be defined as the co-occurrence of lexical items that “appear with greater than random probability” within a specific span (Hoey, 1991, p. 7). *Lexical items* here refer to lexemes. Hence, for instance, *make a decision* and *make decisions* are considered as instances of one collocation. This study took a broad view of collocation which does not distinguish between collocations and idioms. Moreover, this study utilized a criterion from the phraseological approach and paid attention to the syntactic relationship between the constituent words in collocations as well.

This analysis fixes attention on *make/take* + the direct objects collocation for the following reasons. Firstly, verb + noun collocations carry essential information, which is indispensable in communication and frequently used by language users (Gyllstad, 2007). Secondly, compared with collocations made up of more complicated verbs, collocations consisting of common verbs are more likely to be used by beginner learners, thus allowing researchers to observe the developmental patterns of collocation use from lower levels to advanced ones. Thirdly, *make* and *take* are among the most frequently used verbs in the learner corpus.

Based on the discussion above, the present research aims to investigate the following research questions:

How does CEFR proficiency level impact three collocational properties? More specifically, what are the semantic features of the direct object in make/take + noun collocations across CEFR levels? Whether advanced learners use collocations consisting of more difficult and longer noun elements?

## Materials and Methods

### Learner Data

The second release of the large-scale learner corpora, EF-Cambridge Open Language Database (Geertzen et al., 2013; henceforth EFCAMDAT), was used in this study. The EFCAMDAT comprises 1,180,310 compositions submitted by 174,743 language learners as assignments to *Englishtown*, an online English language school (Huang et al., 2018). Learners are from about 200 nationalities, with Brazilians, Chinese, Mexicans, and Germans accounting for 70% of the composition. The proficiency levels of students are validly determined by their performance in placement test when they start or advance to a language course. The EFCAMDAT is a pseudo-longitudinal corpus containing a collection of essays written by learners whose proficiency levels span from A1 to C2 level in terms of Common European Framework of Reference for Languages, which enables the exploration of a general developmental pattern of learners’ collocational knowledge.

Compositions in the EFCAMDAT are elicited by means of writing tasks on a wide variety of topics and graded by teachers. There are 128 different writing activities in the full course which consist of topics, such as editing an online profile, writing to a pen pal, and reporting a news story (Geertzen et al., 2013). These topics help to generate varied situations for eliciting a wide variety of collocations from the learners. Each writing task suggests an expected word count according to the complexity of the topic and learners’ language proficiency, ranging from approximately 30 words in lower levels to approximately 150 words in higher levels.

We randomly extracted 3,600 compositions from A1, A2, B1, and B2 CEFR levels, respectively, for analysis to obtain a manageable data size. Due to the relatively small numbers of essays written by advanced learners, compositions written by learners at C1 and C2 levels were treated as a whole to represent essays written by C level learners, from whence 3,600 scripts were extracted. Table 1 presents a summary of the total number of words of extracted data.

**Table 1:** Summary of randomly extracted learner data.

CEFR level	A1	A2	B1	B2	C1, C2	In total
Number of scripts	3,600	3,600	3,600	3,600	3,600	18,000
Number of tokens	129,058	198,020	259,693	490,921	457,678	1,535,370

### Procedures

The EFCAMDAT data were uploaded to Sketch Engine (Kilgarriff et al., 2014) and tagged with the TreeTagger Tag Set (Santorini, 1990). Firstly, corpus query language (CQL), namely, *[lemma = “make”][]{0,4}[tag = “NN.?”]* and *[lemma = “take”][]{0,4} [tag = “NN.?”]*, was run on Sketch Engine to extract *make/take* + noun sequences. All the retrieved sequences were manually checked to remove the infelicitous or incomplete ones. For example, *take dog* may be extracted from *take care of my dog*, which would be removed as *dog* is not the direct object of *take*.

Secondly, in line with Durrant and Schmitt (2009), the British National Corpus was used as reference corpus to retrieve frequency information of component words within each sequence and the sequence as a whole. We first downloaded the BNC XML edition (BNC XML, available at http://www.natcorp.ox.ac.uk/; BNC Consortium, 2007) and removed its xml tags. It was then uploaded to Sketch Engine for the extraction of *make/take* + noun sequences employing the same corpus query language. Afterward, the retrieved sequences devoid of meaning was eliminated by hand. Ninety percent of the data in BNC consists of written language is extracted from a wide range of registers, such as novels, news, and thesis. It contains a 100 million-word sample of modern British English from the late 20th century which makes it a credible reference source. What is more, the convenient data accessibility and handy XML format have made it an ideal reference corpus in our analysis.

Thirdly, a Python (version 3.7.2) script was written to calculate the *t*-score of each *make/take* + noun sequence found in learner corpus based on their observed frequency in reference corpus BNC. Among the varied kinds of collocational association strength measures, the *t*-score method was selected to identify collocations in learner data. The *t*-score measurement was considered to be one of the major measures of collocation strength and more reliable than other measures, such as the *z*-score (Schmitt, 2010). Following Durrant (2008), *make/take* + noun pairings in our research with a *t*-score higher than 3.9 were regarded as collocations. Table 2 summarizes the number of *make/take* + noun combinations and collocations used by learners across proficiencies. About 61% of the *make/take* + direct noun object combinations were identified as collocations.

**Table 2:** Summary of make/take + noun patterns identified from learner data.

	Category	A1	A2	B1	B2	C	In total
Combinations	*make/take* + noun (token)	134	927	719	1,083	1,208	4,071
	*make/take* + noun (type)	25	50	81	116	119	391
Collocations (*t-*score > 3.9)	*make/take* + noun (token)	68	493	471	723	746	2,501
Retention rate		50.75%	53.18%	65.51%	66.76%	61.75%	61.43%

The following information was annotated to each collocation for analysis. First of all, the UCREL Semantic Analysis System (Rayson et al., 2004) was used to annotate the noun elements in collocation with semantic tags *via* Free USAS English web tagger. USAS is a semantic analysis system based on Tom McArthur’s Longman Lexicon of Contemporary English (Mcarthur, 1981) and reported to achieve a precision value as high as 91% (Rayson et al., 2004). The semantic tags refer to the semantic fields which are collections of related word senses. Word senses were grouped into 21 major discourse fields. The tagged results were manually checked and corrected. The list of semantic fields and their sub-categories is summarized in Table 3 based on the tag set description provided in the USAS (Piao et al., 2015).

**Table 3:** Summary of USAS tag set.

Number	Semantic fields
A	GENERAL & ABSTRACT TERMS
B	THE BODY & THE INDIVIDUAL
C	ARTS & CRAFTS
E	EMOTIONAL ACTIONS, STATES & PROCESSES
G	GOVT. & THE PUBLIC DOMAIN
H	ARCHITECTURE, BUILDINGS, HOUSES & THE HOME
I	MONEY & COMMERCE
K	ENTERTAINMENT, SPORTS, & GAMES
L	LIFE & LIVING THINGS
M	MOVEMENT, LOCATION, TRAVEL, & TRANSPORT
N	NUMBERS & MEASUREMENT
O	SUBSTANCES, MATERIALS, OBJECTS, & EQUIPMENT
P	EDUCATION
Q	LINGUISTIC ACTIONS, STATES, & PROCESSES
S	SOCIAL ACTIONS, STATES, & PROCESSES
T	TIME
W	THE WORLD & OUR ENVIRONMENT
X	PSYCHOLOGICAL ACTIONS, STATES, & PROCESSES
Y	SCIENCE & TECHNOLOGY
Z	NAMES & GRAMMATICAL WORDS

Secondly, the English Vocabulary Profile (Kurtes and Saville, 2008) was employed to annotate noun elements with difficulty levels. The EVP project was initiated to substantiate the vocabulary that L2 learners typically know at different CEFR levels. This project assigns each word in its wordlist a level between A1 and C2 on CEFR underpinned by extensive research on 50-million-word Cambridge Learner Corpus and curricula analysis (Capel, 2010). The EVP was reported to be an effective and promising benchmark (Leńko-Szymańska, 2015). The VLOOKUP function in Microsoft Excel was used to assign EVP levels to noun elements within collocation.

Moreover, to verify whether advanced learners tend to use collocations comprised of more extended noun elements, the LEN function was used to calculate the length of each noun.

## Results

### The Semantic Fields of Noun Elements Within *Make/Take* + Noun Collocations

**Figure 1:**
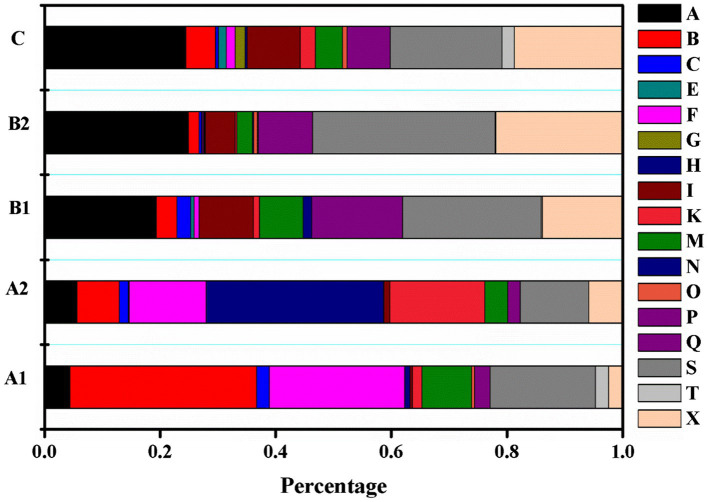
Percentage of nouns in each semantic field across CEFR level.

This section analyzes the semantic features of noun elements in learners’ collocation use. Figure 1 shows the proportion of noun elements belonging to different semantic fields used by learners at each CEFR level. What stands out most is that the percentage of nouns belonging to semantic field A (General & abstract terms), X (psychological actions, states, & process), and I (money & commerce in industry) increased as learners’ language ability improved. In contrast, the number of nouns belonging to the semantic field B (The body & the individual) and F (food and farming) decreased at higher levels. Moreover, the results indicate that noun elements belonging to certain semantic fields are used by learners at relatively higher proficiencies. For instance, nouns within semantic fields G (Government and the public domain) are only used by B2 and C learners; E (EMOTIONAL ACTIONS, STATES, & PROCESSES) are used by learners above A2 levels.

Table 4 displays the two semantic fields accounting for the highest ratio at each CEFR level, as well as frequently used examples from each. Nouns from semantic fields A and S account for the highest ratios among the collocations used by advanced learners.

**Table 4:** Top two semantic fields at each level.

CEFR level	Ratio	Semantic Fields	Examples
A1	19%	F	*make breakfast/dinner/lunch*
	30%	B	*take bath, take a nap, take a shower*
A2	14%	K	*take a break*
	35%	H	*make bed*
B1	20%	A	*make reservation/application*
	24%	S	*take care/part, make friends*
B2	25%	A	*make mistakes, take a risk*
	31%	S	*take part/responsibility*
C	20%	S	*make a difference, make mistakes*
	25%	A	*make efforts/sense*

To better characterize the CEFR levels in terms of the distribution of semantic fields of noun elements, setting learners’ proficiency levels as row variables, and semantic fields as column variables, a correspondence analysis was conducted using R 3.5.1. Correspondence analysis enables the summarization of multiple data sets and visualization of their relationship through a two-dimensional graph (Ishikawa et al., 2010). By plotting the groups of compositions at each CEFR level and semantic features of noun elements together in the bi-dimensional space, we can observe which features could better distinguish each group. This method fits the current analysis as it can deal with categorical data (Ishikawa et al., 2010).

**Figure 2:**
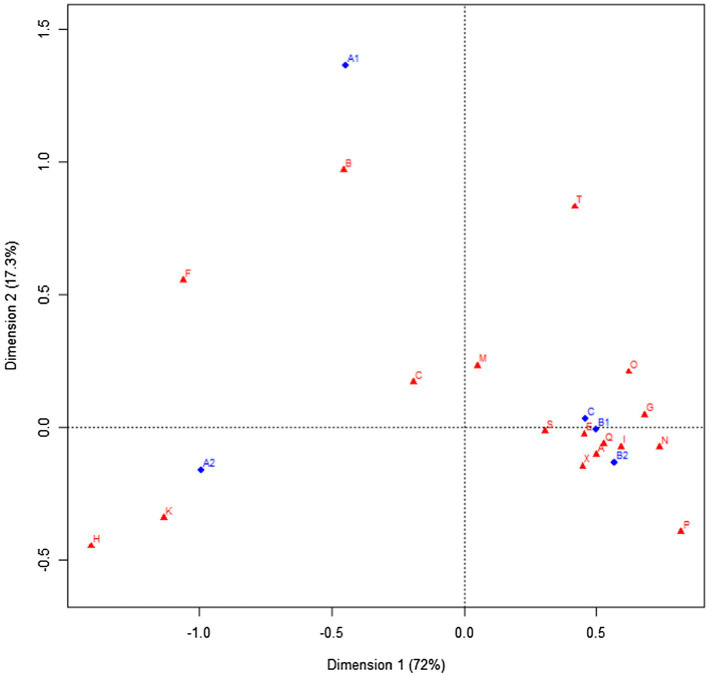
Bi-plot of correspondence analysis: CEFR levels and semantic fields of noun elements.

Figure 2 is the bi-plot of CEFR levels and the semantic fields of noun elements. The cumulative contribution rate of Dimension 1 and Dimension 2 in our correspondence analysis sums up to 89.31%, indicating that these two dimensions explain the variance between CEFR levels and semantic fields to a high degree. To understand the relationship between row and column variables, we can first graph a vector connecting the origin and the plotting point of semantic fields (K, for instance). Afterward, perpendicular line from the position of each CEFR level was drawn to this vector. We need to observe how close each CEFR level is on this vector to the point, K. It can be seen from the bi-plot that A2 is the closest, A1 follows, and the other levels are the furthest. Accordingly, noun elements from semantic field K are most characteristic to A2 learners, and least associated with intermediate and advanced learners. All in all, the bi-plot shows that A1 learners are more likely to use nouns belonging to B (The body & The individual) and F, while A2 learners prefer those from H (Architecture, building, houses, & The home) and K (ENTERTAINMENT, SPORTS, & GAMES). Moreover, the other higher-level learners (B1, B2, and C) were plotted very closely to each other, which shows that their use of noun elements is relatively similar in terms of the semantical features. Learners at these three levels appear to rely on nouns belonging to A (GENERAL & ABSTRACT TERMS), S (Social actions, states, & process), E (Emotional actions, states, & process), and Q (linguistic actions, states, & process), etc.

### The Difficulty Level of Noun Elements in Collocations

To clarify whether advanced learners tend to use collocations consisting of more difficult noun elements, we assigned each noun element with its difficulty information, i.e., EVP level. Nouns annotated with A1 level are supposed to the easiest words, while those with C2 level are the most complicated ones. Goodman and Kruskal’s Gamma coefficient is used to measure the association of the two ordinal variables, *G* = 0.36, *p* < 0.01, indicating a positive relationship between learners’ CEFR level and the EVP level of noun elements. Table 5 presents the adjusted residual scores in our analysis, which shows the difference between observed and expected values for each cell.

**Table 5:** Crosstabulation of proficiency level and difficulty level of noun elements.

CEFR levels	EVP levels of noun elements					
	A1	A2	B1	B2	C1	C2
A1	39 (6.06)	13 (−0.88)	8 (−4.25)	8 (−0.13)	1 (−0.37)	0 (−1.06)
A2	274 (17.28)	139 (2.91)	75 (−10.61)	3 12(−8.74)	0 (−3.61)	2 (−2.31)
B1	103 (−1.89)	111 (0.19)	204 (3.80)	49 (−1.26)	0 (−3.51)	4 (−1.38)
B2	109 (−7.48)	107 (−6.37)	321 (5.76)	163 (10.19)	17 (0.61)	6 (−1.88)
C	107 (−8.18)	211 (3.93)	286 (1.79)	80 (−1.37)	34 (5.67)	27 (5.43)

Adjusted residuals appear in parentheses below observed frequencies.

According to Table 5, the adjusted residual scores of A1 and A2 learners in the use of noun elements at A1 difficulty level are the greatest, while that of C learners are the smallest. It implies that A2 leaners are most inclined to use noun elements at A1 levels, whereas C level leaners are least incline. Meanwhile, the residual scores obtained by C learners in the use of noun elements at C1 and C2 difficulty levels are the greatest, indicating that advanced learners tend to use those difficult nouns most. The residual statistical analysis further confirms the tendency that students with higher English proficiency tend to use more difficult words.

### The Length of Noun Elements in Collocations

**Figure 3:**
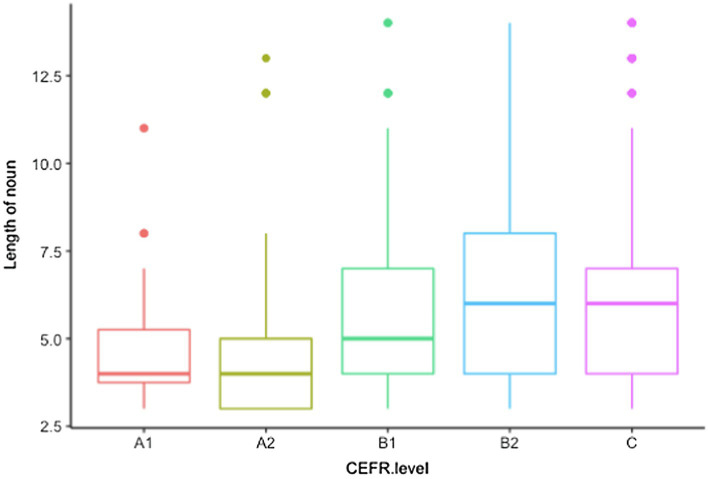
The relationship between the CEFR level and length of noun elements.

The relationship between learners’ proficiency levels and the length of noun elements is presented in Figure 3. As can be seen from the graph, learners’ collocation use at B and C levels contains a greater proportion of noun elements composed of seven or more letters.

We employed mixed-effects models to analyze the contribution of multiple factors to the length of noun elements used by learners. Learners’ CEFR level was set as fixed effect, while individual learner, nationality, and writing topics as random effects.^1^ A1 level was set as the reference level of the categorical predictor variable. The statistical analysis was conducted using the lmerTest package in R (version 3.6.3).

Table 6 presents the parameter estimates from the model. The results indicate a statistically significant difference in noun length between the reference level (A1) and B1 level (*Estimate* = 0.92, *SE* = 0.45, *t* = 2.04, *p* < 0.05). Accordingly, the expected noun length in the B1 level tended to be longer by 0.92 words than that of A1. Moreover, there was a significant difference between the A1 level and C level (*Estimate* = 0.94, *SE* = 0.44, *t* = 2.1, *p* < 0.05), suggesting that the expected noun length is longer at the C level than at the A1 level by 0.94 words. Nevertheless, the analysis found no significant differences between A1 and A2, A1, and B2 level.

**Table 6:** Summary of the mixed effects model for the length of noun elements.

Parameters	Fixed effects				Random effects		
					Topic	Nationality	learner
	*Estimates* (95%-CI)	*SE*	*t*	*p*	*SD*	*SD*	*SD*
Intercept	4.99 [4.24;5.72]	0.40	12.62	0.0001^***^	0.87	0.09	0.0004
CEFR A2	−0.28 [−1.14;0.63]	0.46	−0.60	0.55	–	–	–
CEFR B1	0.92 [0.05;1.80]	0.45	2.04	0.04^*^	–	–	–
CEFR B2	0.82 [−0.03;1.69]	0.45	1.82	0.07	–	–	–
CEFR C	0.94 [0.09;1.72]	0.44	2.16	0.03^*^	–	–	–

* *p* < 0.05;

*** *p* < 0.001.

## Discussion

The present study was designed to provide insights into the characteristics of learners’ collocation use at different proficiency levels. We randomly selected 18,000 essays from the EFCAMDAT and extracted 4,071 *make/take* + noun pairings. *T-score* value, a measure of collocational strength, for each *make/take* + noun pairing was calculated, based on which 2,501 pairings were identified as collocations. Those collocations were annotated with necessary information and then examined concerning the semantic features, difficulty levels, and length of noun elements in collocation. Our focus was on the different characteristics of EFL learners’ collocation use at each proficiency level.

Over half of the *make/take* + noun combinations used by learners at each proficiency level were identified as collocations with *t*-scores higher than 3.9. In terms of the semantic features of noun elements within collocation, the quantitative analysis has found an association between the proficiency level and the semantic fields of noun elements. It was found that beginner learners mainly used collocations containing nouns belonging to the semantic fields B, K, H, and F which were about everyday activities and concrete objects. The advanced learners are found to behave in a similar way regarding the semantic elements they used. They tended to use collocations belonging to semantic fields, such as A, S, E, and Q, which are concerned with abstract social/psychological/political topics. Our analysis has shown that proficient learners tend to use noun elements of higher difficulty levels. Moreover, although there was no significant difference in the length of noun elements used by A1 and A2 learners, B1 and C learners were found to use longer nouns than A1 learners. To summarize, our analysis implies that EFL beginners tend to use *make/take* + noun collocations containing relatively concrete, easy, and short noun elements, while advanced EFL learners manage to combine the common verbs with semantically more complicated, difficult, and relatively longer nouns for various communication tasks. This result aligns with previous research conducted by Namvar (2012), who found a strong and positive relationship between learners’ collocational knowledge and their overall proficiency. In addition, the current analysis also echoes that of Nizonkiza (2012) which suggested that learners’ productive collocational knowledge develops as their proficiency increases.

The present results are significant as it facilitates our understanding of the developmental patterns of EFL learners’ productive collocational competence. EFL learners are widely assumed to focus on the learning of individual words without paying attention to their co-occurring companions (Wray, 2002). However, our analysis has shown that over half of the *make/take* + noun combinations used by learners are native-like collocations in writing tasks. Meanwhile, their collocational competence kept growing until they are able to use collocations containing noun elements of more varied semantic fields, which may enable them to accomplish diversified communication activities. This study has identified that EFL learners tend to try out the combinatorial mechanisms and mimic the combination of words as native speakers do from the early stage of language learning. Our study supports Durrant (2008) and Durrant and Schmitt (2010), who claimed that EFL learners “do retain information about what words appear together in their input” (p. 1) and intensive exposure to collocations can improve their language acquisition. Therefore, the findings can be considered as positive news to EFL teachers as students’ productive collocational knowledge appear to develop as their proficiency grows. In accordance with Boers et al. (2014), we propose that involving learners in extensive exposure to collocations and varied communication tasks would encourage the deliberate learning of collocations and elicit diverse collocations from them.

Our results provide implications for the teaching of collocations as well. We found that the semantic fields of noun elements characterize learners’ collocation use at different proficiencies. Beginner learners’ ability of combing abstract noun elements within semantic fields, such as social action and economics, appears to be underdeveloped. Instructions facilitating the mastery of collocations containing noun elements within such semantic fields can be particularly beneficial to EFL learners at lower proficiencies. With respect to the ideal way of presenting collocations, Lewis (1993) stated that vocabulary organized according to topics or semantic fields leads to a more effective memorization than randomly occurring vocabulary. Meanwhile, Karoly (2005) has also emphasized that learners should record collocations in an organized way. Therefore, we encourage EFL teachers and material compilers to present collocations according to specific semantic fields and have learners acquire them collectively.

The findings also expand the previous work which set out to examine the possibility of utilizing learner’s collocational competence as a possible criterial feature. According to Hawkins and Buttery (2010), criterial feature refers to “linguistic properties that are characteristic and indicative of L2 proficiency at each level, on the basis of which examiners make their practical assessments (p. 2).” It has immediate implications for EFL learners, teachers, as well as teaching material compilers. The present study captures a set of properties characterizing learners’ productive collocational knowledge across CEFR levels. Collocations within semantic fields, for instance, G and E, are used by learners at certain proficiency levels only, which appear to be promising criterial features that could distinguish learners at adjacent CEFR levels. Future studies on the current topic are highly recommended.

However, the findings need to be interpreted with caution for the following reasons. Firstly, the use of nouns in different semantic fields tends to be greatly influenced by the topic of given tasks. Many studies have shown that the lexical choices that language users made differ remarkedly across disciplines, registers, and genres (Biber and Conrad, 1999; Hyland, 2008). L2 learners’ language production is no exception. Alexopoulou et al. (2017) investigated pairs of tasks in three task types, *viz.* narrative, descriptive, and professional. Topics, such as cruise complaints, would elicit compositions with higher linguistic complexity than other topics, such as a job ad. Higher Englishtown-level learners might have been assigned more complicated writing tasks that required abstract nouns for successful completion. Therefore, further research based on the investigation of essays written under the same pair of tasks would offer us more valid information on learners’ collocation. Secondly, it is important to bear in mind that the vocabulary learning mechanism is extremely complicated and dynamic. The increase in learners’ overall proficiency and accumulated learning of individual words might greatly influence them when deciding which words to use in collocations. The investigated properties of collocation use may also be a result of the increase of overall proficiency.

## Conclusion

The present study investigated the properties of learners’ productive collocational competence at each CEFR level. The main findings of this study are that beginner learner is able to use verb + noun collocations consist of nouns concerning concrete objects and daily activities, while intermediate and advanced learners are able to use collocations containing semantically varied and complicated noun elements. Moreover, the results suggested that proficient leaners are to use collocations containing more difficult and relatively longer noun elements in *make/take* + noun collocations. The findings of this study have a number of practical implications on language teaching and the exploration of criterial features. It also provided a time and energy-efficient way of identifying collocations using a programming language based on the observed frequency of the collocations and their constituent words in a large-scale native corpus.

## Data Availability Statement

The original contributions presented in the study are included in the article/supplementary material, further inquiries can be directed to the corresponding author.

## Author Contributions

MA is the main corresponding author of this manuscript and completed the overall write-up of the manuscript. Analysis and discussion sections are conducted by XD. All authors contributed to the article and approved the submitted version.

## Funding

This project is funded by the research supporting project number (RSP-2021/251), King Saud University, Riyadh, Saudi Arabia.

## Conflict of Interest

The authors declare that the research was conducted in the absence of any commercial or financial relationships that could be construed as a potential conflict of interest.

## Publisher’s Note

All claims expressed in this article are solely those of the authors and do not necessarily represent those of their affiliated organizations, or those of the publisher, the editors and the reviewers. Any product that may be evaluated in this article, or claim that may be made by its manufacturer, is not guaranteed or endorsed by the publisher.

## Footnotes

^1^It should be noted that the topic ID used in the present study may not be as credible as it could have been as the original EFCAMDAT data happen to contain texts which did not coincide with the listed task prompt. The EFCAMDAT Cleaned Subcorpus (Shatz, 2020), a derivative corpus of EFCAMDAT, has achieved higher reliability in the annotation of task prompts (topic ID) and can be an ideal option in future analysis.
